# Abstracts and Gleanings

**Published:** 1881-02-20

**Authors:** 


**Treatment of Burns.**—At St. Francis' Hospital, N. Y., Dr. G. F. Shrady is in the habit of using the following dressing for burns and scalds: gum acacia 3 oz., gum tragacanth 1 oz., carbolized water [1-16] 1 pint, and molasses 2 oz. Apply with a broad, flat camel's-hair pencil. This plan of treatment is essentially that of the late Dr. Gurdon Buck, and really has very many advantages.—*So. of the Co. of Kings.*

**Iodoform in Gynæcology.**—Dr. Kurz, in *Allgem. Med. Centralezit.*, February, 1880, states, that he has employed iodoform, and with excellent results, in the treatment of chronic metritis, perimetritis and periuterine phlegmon, as also in ulcerations of the cervix.

In treating such cases, a tampon may be saturated with a solution of iodoform, one part in ten parts of glycerine, and then introduced; or an ointment of the same strength may be used. These applications are much superior to those of tincture of iodine, as they calm the pain, and even sometimes induce a slight degree of general narcosis.

The iodoform tampon should be introduced twice a week, and at the same time inunctions with an ointment, containing one part in ten, should be made over the abdomen.—*Medical Reporter.*

### ABSTRACTS AND GLEANINGS.

**Eczema of the Genitals.**—We extract the following practical suggestions from an article on this subject by Dr. Buckley, in the New York Medical Record:

The most common single, general symptom observed in patients with eczema of the anal or genital region is constipation, or, as it might be more properly called, imperfect intestinal excretion, generally with faulty liver-action; indeed, this almost invariably exists to a greater or less extent, and requires to be looked for and managed properly. So commonly have I found this in the very considerable number of cases of eczema of the anus and genital region which have been under my care, that I had felt that I could almost state it to be an invariable accompaniment of this condition; but, on going over my notes of cases, I find a certain small proportion in whom it is stated by the patients that the bowels acted regularly once or twice daily. This is not, however, convincing proof to me that the intestinal action was perfect, and I still believe this to be the most important single factor in the disease. Quite possibly the irritating character of the excrement itself is an efficient local cause of the presence and continuation of the eruption.

This imperfect intestinal excretion should be corrected, if possible, and very great care will sometimes be necessary to accomplish this. It is not enough to give occasional purgatives, nor even to prescribe daily laxatives; for, unless much caution is exercised, the ultimate result in this direction may be bad instead of good. These remarks in regard to the management of this important element may seem trite and out of place before this learned body, but I wish to impress the very great importance of dealing with this portion of the treatment rightly as a *sine qua non* of the successful management of eczema of the parts under consideration.

All the elements which conduce to bring about a healthy action of the bowels and organs of digestion must therefore be attended to, and, consequently, in the treatment of eczema about the anus and genitals we must not be content with a few general directions, or the prescription of one or another purgative or laxative remedy. On the contrary, it may require no little trouble to ensure a healthy evacuation of the bowels daily, and this is accomplished by diet, exercise, regularity in attending to the call of nature, and such assistance from medicine as may be necessary.

A very common accompaniment of eczema of the regions under consideration is a greater or less congestion of the portal and hemorrhoidal circulation, manifested by a purplish congestion of the mucous membrane of the anus, or very commonly by a greater or less degree of internal or external piles. These latter may not be sufficient to be recognized by the patient, and yet be an element indicative of the existing state which must be regarded. It is well, therefore, in examining patients thus affected, to have them strain or bear down to bring the deeper portions to view.

When this congestion of the hemorrhoidal vessels exists I almost invariably give the time-honored mixture of precipitated sulphur and cream of tartar, in quantity sufficient to secure one or two loose movements from the bowels daily. I never give it with syrup, as I believe this often ferments or acts prejudicially in the stomach, and in a measure impairs the good effects. I order a mixture of bitartrate of potassa in equal quantities, and direct that from one to two teaspoonfuls be taken at night on retiring, rubbed up with water into a paste. The dose is not a very pleasant one, but it is readily taken, even by ladies.

Where there is no marked hemorrhoidal congestion I employ a pill of two grains and a half each of blue mass and compound extract of colocynth, with a quarter of a grain of powdered ipecac in each pill; two such pills to be taken at night and two on the second night after, followed each morning by a seidlitz powder or Kissingen water. These pills are to be taken only twice, and are not resorted to again at a less interval than a week or two; but they may be thus used repeatedly with good effect.

If there is simply a sluggish action of the bowels. a grain of the extract of socotrine aloes with a grain of dried sulphate of iron and a little aromatic powder and confection of roses, one pill being taken directly after eating. Very much may be accomplished by this combination in the way of permanently overcoming the constipated habit if the pills are employed regularly and systematically according to the following directions: at first one pill is taken directly after each meal, three times daily; in a few days the noon pill is omitted, and a few days later one is taken after the evening meal only, and soon this is required less frequently, and subsequently omitted. The point to be insisted on is that the pills shall be used regularly in the above manner until the bowels acquire the habit of daily excreting and discharging a normal amount—if they are taken irregularly simply for a cathartic action, no ultimate good results follow; but I can bear testimony very strongly to the value of this plan of treatment, and could adduce many cases where this has constituted one of the chief means of speedy and permanent cure of long-standing cases of eczema of the anal and genital regions.

If internal and general measures are important in eczema of the anus and genital region, local measures are, if possible, of even greater importance; it is much not to do the wrong thing, and still more to do just the right thing. This remark is made because one occasionally sees cases which have been greatly aggravated by previous treatment, which yield promptly to proper measures. The main point to be ever borne in mind in the treatment of these parts is that more harm than good may be done by too strong applications, and that the soothing plan must be followed as far as possible, certainly while there are signs of inflammation, stimulating measures being adopted only in later stages of treatment, and to remove the remains of the disease, as thickening of the skin, and not for the arrest of the eczema.

The itching of these cases is often most intense, and the patient will plead that if he can only have something to stop the itching the disease will get well. And so I have repeatedly had cases where all sorts and kinds of measures had been previously prescribed with a view of arresting the itching, but in vain, whereas the case yielded speedily when complete treatment was instituted, including only very mild local mesasures. Quite recently a physician brought a patient in consultation, not in regard to any general management of the case, but only to have my opinion in regard to the probable utility of applying the actual or galvanic cautery to the parts to arrest the itching. And so I have had cases which had previously been given stronger and stronger local applications, with a view of checking the itching, after the failure of recognized neurotic local remedies, until the parts had been brought to a terrible state of inflammation from such applications as strong citrine ointment and the like. Now, while these may succeed in some cases in which, perhaps, a transient, digestive disturbance was the starting point of the eczema, I am confident that in the main all such attempts in the way of a local treatment of eczema in these parts is false in theory and injurious in practice.

The measures which I am about to detail may be simple, but will in most, if not all cases, be sufficient as local treatment, provided that all else has been carefully attended to as implied in the preceding brief mention of dietetic, hygienic and internal medication.

I place great reliance upon hot water as a means of relieving the congestion of the parts and the consequent itching. But the water should be indeed hot, and not warm—so hot that the hand cannot be thrust wholly into it—and it should be used in exactly the manner now to be described. I speak thus positively because I occasionally hear it asserted by patients that it is not of service, and on inquiring I find that the exact rules have not been followed, or that it has been used for a longer time or oftener than prescribed. The patient should sit on the edge of a chair and have a basin with the very hot water and a soft handkerchief in it. This latter is then picked up and held in a mass to the anus or genital parts, as hot as can be borne, say for a minute, and then dipped in the water again, and the process repeated three times, the whole not lasting more than two or three minutes; too long bathing, or too frequent sopping of the part or rubbing with the cloth, etc., makes matters worse.

Before the hot water is gotten ready, I have the ointment which is to be employed spread thickly on the woolly side of surgeon's lint, cut of a size to cover the affected parts only, and laid close by ready for immediate use. After the parts have been soaked with the hot water for the preseribed time, they are rapidly dried by pressing a large, soft linen napkin upon them, with absolutely no friction, and the already spread cloths are immediately applied, the object being to at once exclude the air entirely. Ordinarily it is necessary to use the hot water only a single time in the twenty-four hours, namely, after undressing, and when ready to get into bed. It must be premised that the patient is to so manage as not to indulge in the usual scratching before undergoing these manipulations. If this desire is given away to beforehand, the treatment will not always control it at once; but if the patient can avoid even touching the parts except as described, he or she will commonly be quite able to go to sleep immediately. I have repeatedly had those thus afflicted say that the first night of treatment was the first real rest they had had for months or years.

If the case is very severe, and if there are spells of recurrent itching, the hot water may be repeated occasionally; but it is commonly sufficient simply to renew the ointment one or more times in the day, especially in the morning on rising, without the repetition of the hot water, which latter, I think, sometimes acts prejudicially in softening the parts if used more frequently. It should be added that the ointment should always be spread on lint and never be rubbed to the part; also, that in applying the lint it should be kept in close apposition to the diseased surface, and that by means calculated to heat the parts as little as possible; and finally, that in renewing the dressing the fresh cloth should be spread and ready, near by, before removing the previous one, that the access of air to the parts may be prevented by changing the coverings as quickly as possible.

The ointments employed must vary somewhat with the case, and no single one could be mentioned which would be invariably of service. That which I most commonly prescribe is made as follows:

℞ Unguent. picis .......................................... ℥j.

Zinci oxidi. .......................................... ʒij.

Unguent. aquæ rose (U. S. P.) .......................... ℥iij.

M.

This should be of a consistence which spreads easily and remains soft, which may be easily regulated by varying the proportion of the spermaceti in the rose ointment or cold cream. I may add that I never employ the recent products of petroleum, cosmoline and vaseline, as a basis for these ointments where protection of the surface and exclusion of air is desired, as they have not body enough to remain as a thick coating upon the limb, but rapidly soak in and leave the parts dry and exposed.

I will not occupy time with further details of ointments, as this is sufficient to indicate the plan or idea of treatment which I wish to present as offering success in the class of cases under consideration; while the ointment is not a matter of indifference, the same result can be obtained I believe by other remedies than the one mentioned, and my case-records would undoubtedly show many others of value. It is the method of employing remedies and strict attention to details which gives success, and I feel certain that the points I have given are very important and will be of the greatest service if carefully carried out.

Brief mention might be made of other applications which have rendered me good service, although, as before remarked, remedies must vary for different cases, and it is beyond the limits of the present paper to detail all that might be used and to give their possible indications. The following combination is very effective:

℞ Unguent. picis.......................................... ʒiij.

Unguent. bellad .......................................... ʒij.

Tinct. aconit. rad.............. ....... ............ ʒss.

Zinci oxidi.......................................... ʒj.

Uuguent. aquæ ros .......................................... ʒiij.

M. Ft. ung.

The ointment of chloral and camphor, of each a drachm or two to the ounce, will often prove a very efficient anti-pruritic, as first described by the present writer several years ago.

Lotions are sometimes of much service, especially in eczema of the penis and scrotum, and the following can be recommended:

℞ Bismuth subnitrat.......... ............ .................. ʒ ij.

Acid. hydrocy, dil........................................ ʒ j.

Emuls. amygd............................................ ℥ iv.

M. Ft. lotio.

This of course must not be used where the skin is much torn or broken.

A word may be added in regard to the employment of stronger local measures, for they are not infrequently of value in proper cases and at the proper time or period in the disease. When congestion has ceased, and there is still some thickening and a tendency to slight cutaneous fissures, we may use the green soap or the compound tincture of green soap.

℞ Saponis viridis,
Olei cadini,
Alcohol........................................ aa ℥ j M.

With good effect. With this we need friction, and a piece of muslin (subsequently white flannel may be used to give greater friction) is wet with the lotion and rubbed briskly over the parts for a few moments, which are then to be immediately covered with a mild ointment. For this purpose the ordinary zinc ointment, half a drachm to the ounce of the unguentum aquæ rose (U. S. P.), answers well, or the subnitrate of bismuth, or calomel, either in the same strength.

**Vaccine Virus.**—Dr. Burge, in a review of discussions, in Medical Society of the County of Kings, says:

In placing my views on record regarding this subject, I ought, perhaps, to say that they are the result of reflection and observation in the ordinary experience of a thirty years' practice, and not the conclusions of an expert, for I have never engaged in any experiments in this line of investigation, and have only the sources of information which are open to all of you. Indeed, I should never have thought of bringing the subject before you again if expressions which I believed to be dangerously false had not gone out to the world from this Society, and been thus far unchallenged. Please recollect that I have had no thought of presenting a general treatise on vaccination; and now allow me to epitomize, in order that no one may mistake the purport of these remarks:

1. I have the utmost confidence in vaccination.

2. I believe humanized virus retains its virtue from generation to generation.

3. I have no faith in any artificial process for obtaining virus.

4. The natural cow-pox is the only source to which we should resort when, for any reason, we need a new supply.

Dr. Ephraim Cutter, who is excellent authority, says: "The natural cow-pox is enzootic in this country, and only needs looking up to be discovered."—*Trans. Am. Med. Assoc.*, 1872, *p.* 235.

5. If lymph *only* be used, it can never convey any disease but vaccinia. This, however, does not obviate the necessity for eternal vigilance against accidental admixture, by which primary syphilis, diphtheria, erysipelas, small-pox, etc., etc., may be communicated.

6. Do not understand me as saying that all the cases that have been reported as syphilitic, after vaccination, have been cases of primary infection.

It is a well-settled fact that a *pure* vaccination is often the exciting cause of great disturbance, and of serious manifestations of disease, in those of syphilitic and scrofulous diathesis.

7. Let nothing that I have said be so construed as to convey the idea that there is no protecting power against small-pox in any of the varieties of vaccine virus now in use. So far am I from entertaining this view, that until I can get lymph directly from the natural kine-pox, or in a direct line of human succession from it, I shall use the calf to calf virus, as the next best thing.

8. I honor the gentlemen who, at great pains and expense, have endeavored to supply the profession with virus which they believed was pure and efficient; and though I question its efficiency, and counsel a return to the old and sure method, I shall be glad to see the evidence that calf to calf virus is as good as that from original vaccinia. The burden of the proof lies with the advocates of the new product, and I for one shall look for it with deep interest. If they make out a good case, I shali be both delighted and surprised.

**Treatment of Phthisis.**—Numerous cases of phthisis are always to be found in the wards of Bellevue Hospital, and various plans of treatment are being pursued with a view to ascertain their comparative merits. Some of the results may be of interest to your readers. For the purpose of aiding in the assimilation of food, it is often necessary to use a bitter tonic before meals to excite an appetite, and with that object a teaspoonful of a mixture of Tr. Cinchonæ Comp. and Tr. Gentiani, equal parts, is given, iron in the form of Tr. Ferri Chloridi is used after meals, and various emulsions of cod liver oil are largely employed. Trommer's extract of malt is found to be of service in many cases. The cough is probably the most distressing symptom, and requires constant treatment. In some cases it is harsh, and expectoration is chiefly to allay the irritation in the bronchi and trachea. This is done by the use of hydrocyanic acid, combined with bromide of potash and given in syrup pruni. virgin, or by mixing equal parts of cyanide of potash and sulphate of morphia in syrup of tolu in such proportion that a drachm of the syrup shall contain one-eighth of a grain of each of the salts. It is not seldom found that a cough of this character is aggravated by an elongated uvula or by an irritable condition of the pharynx. If so, the pharynx and the uvula may be painted daily with a solution of nitrate of silver, gr. lx. to ℥ j. with the result of allaying the irritation, or the extremity of the uvula may be clipped off with scissors. When laryngeal irritation is the chief factor in the cough, the use of narcotics is indicated, and a favorite mixture is one composed of equal parts of Hoffman's anodyne and U. S. sol. of morphia. This may be given in doses of ʒ j. to ℥ ss., and many patients find it more serviceable than any other combination in arresting the cough at night and inducing sleep. To relieve irritation and excite secretion in a dry catarrh, the wine of ipecac is used in the form of a spray, and with a little care it is possible to apply it to the greater part of the larynx, and even to reach the trachea, if the atomizer is properly directed, and used during the act of inspiration. All inhalation of powders has been abandoned in favor of the spray. When the cough is loose and expectoration free, those drugs are combined which will lessen the tenacity of the mucus and increase the power of the bronchial muscles. Stoke's expectorant mixture, containing Ammon. Carb., Ext. Senegæ, Ext. Scillæ and paregoric with syrup tolu is largely used, and a combination of Carbonate of Ammonia, ℥ j. with Spts. Chloroformi ʒ jv. and Infus. Cascarillæ ℥j, of which the dose is ℥ ss., has also been of service. Some one of the above cough mixtures is usually employed with success, the indication to be met being ascertained before any selection is attempted.

The night sweats of phthisis are so weakening in their effect that some measures have to be employed to check them. A tepid sponge bath containing alum may be of service when the sweats are beginning and are not very severe. Sooner or later recourse must be had to some preparation of belladonna, and a simple solution of atropia is as efficacious as any elaborate combination. Of late, nitrite of amyl in doses of three drops given on a lump of sugar has been found useful, and it may be employed without or with a small amount (1-100 gr.) of atropia with good results. In several cases when atropin alone has failed to check the sweating unless given in doses large enough to produce disagreeable effects, it has succeeded when in combination with the nitrite of amyl. In other cases, the amyl alone is all that is needed. Various theories of its action are proposed, but no one is quite satisfactory. The fact of its efficacy is not disputed. The use of alcohol in phthisis has been sufficiently debated in books and journals. In the hospital it is at present freely used in most cases, and, it is thought, with good results. In the treatment of the diarrhœa of phthisis, opium is chiefly relied upon. The deodorized tincture, McMunn's Elixir, or a powder containing 1-6 grain of morphia, and 10 grains of bismuth are used indifferently, though the latter is probably employed in the majority of cases. To control the febrile movement nothing can supersede quinine.—*Cor. Chicago Medical Review.*

**Pneumonia.**—Dr. Corson in *Medical and Surgical Reporter*, says of the treatment of pneumonia :

To me it is simply an inflammation, and I would use rapidly, as early as possible, the means which, in a practice of half a century, I have found safe and most efficient in allaying inflammation. Well, here is my patient; he had a chill hours ago; he has some pain in his side, some cough, he feels very weak, his face is flushed, he is hot and theisrty, his pulse is over a hundred, the thermometer rises to 103°, he breathes scarcely at all through most of one lung; there is a little blood and viscous fluid, slightly rust colored, in the basin by the bedside: he knows how he got sick; he "was down in the iron ore shaft all yesterday, at work; came up in the evening to come home, and being in a perspiration, soon, in facing the wind, cooled off too suddenly." I propose to bleed him; he objects, on account of his great weakness. I tie up his arm, bleed him twenty ounces, or perhaps he feels faint by the loss of less than that; expresses himself as being relieved somewhat of his oppression ; he can lie more comfortably, he feels relieved of the fullness in the chest, but still he has a little pain in the side, or in the breast just above the nipple. I order him one-quarter gr. sulph. morph., direct that a towel wrung out of cold water, ice-water, if convenient, be applied over the affected part,to be changed frequently—or substituted by a bladder of ice and water. Then directing a mixture which contains some spirits of nitre, with about the 32d of a grain of sulph. morph. to teaspoonful every two hours, I leave him, with an express direction not to worry him by any offer of food. Next day, or even the same day, perhaps if I find that he is not entirely relieved of the pain, and is still expectorating viscid sputa, I bleed him again freely, and if need be, cup him, and after that he is generally safe. Some one will say—that may do very well for young, strong iron-ore diggers, but it would prove fatal to our delicate patients. Perhaps so, but in my experience I have found that the weak and frail need to have the same means used to allay a toothache or a sciatica, a pleurisy or a rheumatism, as the robust. They bear the remedies to cure an ague as well, and need them as greatly, as the strongest. I have often bled the infant strangling with croup, with immediate relief; weak women with pleurisy or pneumonia, old men and aged, very aged women, to relieve affections of the brain, or avert a paralysis.

**Danger of Uterine Manipulations and Operations.**—Dr. G. J. Engleman, in a paper to the State Medical Society of Missouri, illustrates the danger of intra-uterine injections. He mentions several cases of peritonitis and death from intra-uterine injections of iodine, and one in consequence of an application of tincture of iodine to the cervix. He says: "Dr. Theophilus Parvin, of Indianapolis, relates a very similar case; his patient was a married woman, 35, sterile, who was suffering from hemorrhage due to uterine fibroids, which she was known to have had for twenty years, At the time Dr. Parvin was consulted the hemorrhage was violent and uncontrollable, persisting, even after free dilation of the cavity and tamponing of the *os uteri;* other means having failed, he injected very freely into the uterus a warm solution of muriated tincture of iron, one part to seven parts of water; the patient at once fell into a collapse, which for half an hour was death-like; she rallied, to die within less than a week of the metro-peritonitis which followed.

Dr. Skene, of Brooklyn, writes me that he has in eleven cases seen violent uterine colic and shock follow the careful injection, into the uterine cavity, of tincture of iodine, water, mild solutions of nitrate of silver, and, in one instance, a metritis, from which the patient was years in recovering, after an injection of less than ʒss—30 drops of equal parts of tincture of iodine and opium.

These cases are but a few of the many, and, notwithstanding all that may be said to the contrary, the injection of fluid into the uterine cavity is a dangerous proceeding; and neither the double canula, or the syringe with gutter, or any of the other ingenious instuments which have been devised to facilitate the exit of the injected fluid are sufficiently reliable in their action to make this method a safe one.

Fischer, of Magdeburg, in an inaugural thesis, which appeared in

Halle A. S. in 1879, has compiled fifty-four published cases of alarming as well as fatal results following intra-uterine injections. It is the injection of the cavity of the undilated uterus which is fraught with danger, and which is uncalled for, since so many other equally efficacious and less dangerous methods of treatment have been devised. The injection of the puerperal uterus (post partum or post abortum), though not absolutely without risk, is so invaluable a remedy, be it hot water in post partum hemorrhage, or the carbolized solutions in puerperal affections, that we must overlook the very slight dangers accompanying them; it is only against the injection of perchloride of iron for the relief of post partum hemorrhage that I would protest, as very dangerous, and, if anything, less efficacious than the iron swab or the hot water douche.

[He mentions the case of Mrs. T. who, while using a syringe, had been seized with a sudden and severe pain—a uterine colic—which was followed by instense suffering.]

"I found my patient in great agony, the abdomen somewhat distended and exquisitely sensitive to the touch, most especially in the region of the uterus and the ovaries; pulse rapid and small; spasmodic increase of the pain. The subcutaneous injection of morph. sulph., gr. 1/6 gave but little relief. Hot applications to the abdomen, and opium in 1/4 gr. doses, slowly overcame the pain, and after midnight she fell into a restless slumber. The following day she felt sore, and remained quietly in bed. A speedy recovery followed.

Unquestionably, a few drops of the injected fluid entered the uterus. Since this time I always close the central orifice of the vagnial nozzle, and direct the injection to be used in the recumbent or semi-recumbent position, as more comfortable for the patient, less tiresome, and more advantageous, as securing a more thorough washing of cervix and vagina, which in this position, can retain the fluid. The habit of sitting or stooping over a vessel is exceedingly tiresome and trying, often, indeed, injurious, and at once neutralizes many of the good effects of the injection.

In order to obviate the dangers and discomforts arising from vaginal injections as ordinarily used, I advise my patients: 1. To plug the central opening of the vaginal attachment. 2. To assume the semi-recumbent, better the recumbent, position, with knees drawn up. 3. fountain or the bulb-syringe."

[He gives six cases of deaths following the use of sponge tents.]—*Medical Abstract.*

**Chloral in After-pains.**—The following case is reported by Dr. Julia Carpenter. She says: I remained with the patient three hours after the birth of the child. During this time there were some after pains, which toward the last were severe enough to require relief, so the following was prescribed:

℞ Morphiæ sulphat., .................................... gr. ss
Aquæ camphoræ, .................................... ℥ j.

Sig. Teaspoonful every half hour, if needed, until three doses are taken.

Called again at eight o'clock p. m. Patient had had several pains every fifteen minutes, but at that time they seemed to be getting under control. At one o'clock at night they came for me, saying the paroxysms were so violent they were alarmed; all the medicine had been taken and still no relief was afforded.

Knowing that the good recovery of this patient depended on the amount of nourishment she could take, no more remedies were given per os. Suppositories of one grain of opium were substituted, and flannels wrung out of hot water were constantly employed, a fresh one with every pain. This would always check a pain, but not prevent its recurrence. I remained with the patient until nine o'clock in the morning, when she seemed to be gradually getting relief. On returning at three o'clock, I found, though four suppositories in all had been used, and hot flannel constantly applied, the pains had returned with great violence.

I now concluded to try the efficacy of a small dose of chloral, without the patient's knowledge, as she had asked not to have it given to her, and by enema, twenty-five grains of chloral, in half an ounce of glycerine and water, were taken. The relief was *instantaneous.* "Oh! I'm so comfortable," was her immediate expression. Not one more pain returned, and the following night was spent in sound and refreshing sleep.

During the hours of severest contractions the flow ceased almost entirely, and no clots passed at any time. Twenty-four hours after the birth of the child the uterus was more than half way below the umbilicus, and subsequent to the relaxtion from the chloral, there was a barely appereciable increase in size. The patient made an excellent recovery.

**Poisoned by Vegetables from Poisoned Soils.**—As it has been published in several newspapers and periodicals during the year that individuals had been poisoned and several had died from eating certain vegetables, such as potatoes and melons, which had been grown in soils where Paris Green had been used too freely for the purpose of killing certain bugs which infested the vines, it would be interesting and instructive to be informed by chemical analysis, or otherwise, how far or to what extent the soil of the ground is capable of eliminating from and imparting to vegetable productions poisonous substances deposited into the earth, either to destroy vermin or to fertilize the ground.

In one of our public institutions, about a year since, it was discovered that a bloody flux was prevailing extensively among its inmates, which prompted the managers of said institution to employ the service of a chemist to ferret out, if possible, the cause of that terrible malady. In his analysis, that chemist was successful in discovering raw and unchanged night soil deposited in the cells of the vegetables grown in the garden of that Institution, and of which the inmates had freely eaten, which had produced the bloody flux of which so many suffered. The night soil had been put in that garden in its natural and crude state, and in large quantities. The ground could not digest it.

As a sanitary precaution not only to public institutions, but to all citizens who purchase vegetables in our public markets, ought not this to be thoroughly investigated? It may be that some gardeners, through ignorance, use too freely unmanipulated night soils, or other substances which are deleterious to the production of wholesome vegetables, and the maintenance of good health.—*Independent Practitioner.*

**Delirium Tremens.**—Mr. A. P. Hayne, of the "Home of Inebriates," San Francisco, Cal., says:

"In all forms of the acute variety there is no combination which, as the result of an experience of many years, can be compared to that of chloral hydrate and one or other of the bromides—especiallly the bromide of potassium. In doscs of twenty grains of the former, with thirty or forty of the latter, given at proper intervals, either with or without a small quantity of spirit, or ale and porter, it is far superior to any other combination we have ever tried. The second or third dose seldom fails, in the majority of cases, to fulfil the main object of our endeavors, viz: to tranquilize nervous excitement, quiet the mental agitation, and produce sleep.

"In the selection of the former of these medicines (chloral hydrate) it is highly important to use none but the best article. We always use imported, generally the *German*, but the English and French are, perhaps, equally good. The American is decidedly inferior and often impure. It should be clear, semi-opaque, and crystaline, with a strong, pungent odor of chloroform, perfectly white.

"It is also an important point that both of these remedies should be given in full doses, otherwise we may be disappointed in their action, either singly or in combination.

"Occasionally we will meet with patients who will resist very large doses, and in these we sometimes combine one-eighth to one-quarter grain of morphine with each alternate dose.

"In an experience of many years, we have generally found the simple plan thus outlined answer all purposes; and while in some instances we have been compelled by the urgency of the case to push these remedies to an apparently alarming extent, sometimes as high as two hundred to two hundred and fifty grains of chloral hydrate in twenty-four hours, we have never seen a fatal result which should be attributed to an overdose of that much abused but invaluable remedy.

"That sudden deaths do sometimes occur in violent attacks of acute alcoholism, in all its forms, is a well-known fact; but in the vast majority of these the post-mortem examinations have revealed the true cause of death, and the explanation is rendered conclusive by the presence of serious effusions, cerebral hemorrhage, or embolism.

"Besides the effects of the chloride-bromide combination which we have just noticed, there is another advantage which the former remedy possesses in stimulating the appetite and enabling the stomach to retain nourishment. It also checks the profuse diaphoreses, controls the delirum, equalizes the cerebral circulation, and is the most certain of all hypnotics."

**Apparent Death as a Result of Asphyxia.**—Medical journals occasionally inform us of wonderful resuscitations brought about by the persistent use of artificial respiration; but two, lately reported to the Academy of Sciences, Paris, by Dr. Fort, Professor of Anatomy in the Ecole Pratique, are especially noteworthy, and teach us to persevere in any efforts we make to bring back signs of life. In the case of a child three years old, who had already been placed in the shroud, Dr. Fort commenced the use of artificial respiration, and after three and a half hours of steady work the child was brought back to life. The other case was that of a drowned man, who had been under water for twelve minutes before the body was recovered. Artificial respiration was commenced an hour afterwards, and after being kept up for hours the man was restored to life. The report of the meeting of the Academy does not give the details of the child's case before the appearance of the apparent death, nor what prompted the doctor to commence the artificial respiration.—*Medical Press and Circular.*

**Toothache.**—Now, a word as to a few medicaments and stoppings, adapted to giving present relief and temporary protection to an aching or sensitive tooth that may be saved. Every physician who treats teeth should have at hand the following:

Creosote and morphia,.................................. 0.05 to 4.5
Clove oil.................................................... 0.05 to 4 0
Bottle of sandarac varnish,
Carbolic acid; dissolved crystals,
A package of gutta percha pellets.

I am strongly averse to advising any physician to have arsenic in readiness for use on teeth. It might, in rare cases, be used with advantage by skillful hands, but I am very sure that in the hands of any but a most competent and skillful dentist, it is an agent far more likely to be potent for evil than good. You had better leave arsenic as an applicant to the teeth in the hands of dentists only, and they had better use it but rarely. For my own part, whenever I find it necessary to extirpate a pulp, I much prefer giving an anæsthetic, and doing the operation quickly and at once with the dental engine, rather than use arsenic.

In this connection, I would remind any surgeon whose eye may follow this communication, of a fact he has seen before . that the dental, engine, with its saws, bitts, burrs, and trephines, is capable of great usefulness in operations upon bone; in cases of necrosis, some resections, ununited fracture, overriding fragments, removal of tumors, etc., and any surgeon may find it occasionally of great advantage to call this instrument into requisition.

Dr. W. E. Garretson, of Philadelphia, has written recently a monograph syecially upon this subject.—*G. Newkirk, M. D., in Chicago Medical Jouanal and Examiner.*

**Treatment of Acute Articular Rheumatism by Subcutaneous Injections of Ergotin.**—Dr. Chevallerean read, at a recent meeting of the Societe Clinique de Paris (*La France Med.*, 1880, p. 724), a communication on the use of ergot in acute articular rheumatism. Having been called to see a little girl suffering with prolapse of the rectum, in whom he desired to use hypodermic injections of ergotin, he found her so crippled with rheumatism that she could hardly be turned on her side to permit the injection to be made. Forty centigrammes of the *solution Yvov* of ergotin having been administered by the hypodermic syringe, next day the rectum continued in position, and, to the doctor's surprise, the rheumatism was much better, and the patient recovered without further medication. A little later, being called in to see a young lady of twenty-three, who was suffering with severe acute articular rheumatism, confined to the joints of the right side, fifty grammes of the solution were injected, with the result of marked amelioration. The injection being repeated three days later, the patient was completely cured within ten days. This treatment was subsequently employed in a number of other cases, and always with success. The cases were not accompanied by high fever.—*Times.*

**Diphtheria.**—Dr. Cleave, (in *Louisville Medical News,*) says:

Diphtheria is a constitutional disease with local manifestations. I regard it as a very important step to get rid of the patches deposited upon the fauces and mouth. To remove all of these promptly, apply with a mop saturated with turpentine. This article penetrates through the tough deposit, lifts it off, and leaves a red, shining base, that very soon gets well. Apply every two or three hours until every vestige of this dirty white deposit disappears and fails to return. I give my patients tinct. iron largely, with quinine and the best old whisky freely, with an ample supply of liquid nourishment. Husband the strength of the patient. No purgatives save as a dire necessity. I regard the turpentine worth more than all other local applications. I still use chlorate of potassium, but doubt its real value. I do not say this plan will cure every case, but I do say it is wonderfully successful. I have been using it successfully for years.

**Reprehensible Practice.**—A correspondent of the *Druggist's Circular* writes as follows: "A few weeks since, a young man suffering from toothache asked a druggist for something that could relieve him. The druggist dissolved eighty grains of chloral hydrate in one ounce of syrup of ginger, gave one drachm of it, undiluted, to the patient, and wrote on the label, 'A teaspoonful every half-hour till relieved.' The patient had six doses as directed, when he was taken with convulsions, palpitation of the heart, and finally unconsciousness, attributed by regular physicians, then called in, to an overdose of chloral. The consequence was that the patient was sick for two weeks from the effects of the dosing, and claims that the druggist did wrong and should pay the doctors' bill. The druggist claims that he was not wrong, and should do the same thing again. Is the druggist culpable or not?"

The editor replies at once, and plainly, that the druggist is guilty of criminal imprudence; and we are convinced that our readers will agree with him in this opinion.

**Chloral Cure for Toothache.**—Dr. Sporer, of St. Petersburg, uses chloral hydrate in the following manner:

Take three or four small lumps of chloral, wrap them in a little wadding, place this tampon in the hole in the tooth, and let it remain until dissolved. The most severe toothache will disappear in a few minutes under this treatment.

